# A simple electronic medical record-based predictors of illness severity in sepsis (sepsis) score

**DOI:** 10.1371/journal.pone.0299473

**Published:** 2024-06-26

**Authors:** Alex M. Cressman, Bijun Wen, Sudipta Saha, Hae Young Jun, Riley Waters, Sharan Lail, Aneela Jabeen, Radha Koppula, Lauren Lapointe-Shaw, Kathleen A. Sheehan, Adina Weinerman, Nick Daneman, Amol A. Verma, Fahad Razak, Derek MacFadden

**Affiliations:** 1 Temerty Faculty of Medicine, University of Toronto, Toronto, Toronto, Ontario, Canada; 2 Division of General Internal Medicine, University of Toronto, Toronto, Ontario, Canada; 3 Li Ka Shing Knowledge Institute, Toronto, Ontario, Canada; 4 Unity Health Toronto, Toronto, Ontario, Canada; 5 Department of Family and Community Medicine, Temerty Faculty of Medicine, Toronto, Canada; 6 Division of Psychiatry, The University of Toronto, Toronto, Ontario, Canada; 7 Sunnybrook Health Sciences Centre, University of Toronto, Toronto, Ontario, Canada; 8 The Ottawa Hospital Research Institute, Ottawa, Ontario, Canada; Universitair Kinderziekenhuis Koningin Fabiola: Hopital Universitaire des Enfants Reine Fabiola, BELGIUM

## Abstract

**Objective:**

Current scores for predicting sepsis outcomes are limited by generalizability, complexity, and electronic medical record (EMR) integration. Here, we validate a simple EMR-based score for sepsis outcomes in a large multi-centre cohort.

**Design:**

A simple electronic medical record-based predictor of illness severity in sepsis (SEPSIS) score was developed (4 additive lab-based predictors) using a population-based retrospective cohort study.

**Setting:**

Internal medicine services across four academic teaching hospitals in Toronto, Canada from April 2010—March 2015 (primary cohort) and 2015–2019 (secondary cohort).

**Patients:**

We identified patients admitted with sepsis based upon receipt of antibiotics and positive cultures.

**Measurements and main results:**

The primary outcome was in-hospital mortality and secondary outcomes were ICU admission at 72 hours, and hospital length of stay (LOS). We calculated the area under the receiver operating curve (AUROC) for the SEPSIS score, qSOFA, and NEWS2. We then evaluated the SEPSIS score in a secondary cohort (2015–2019) of hospitalized patients receiving antibiotics. Our primary cohort included 1,890 patients with a median age of 72 years (IQR: 56–83). 9% died during hospitalization, 18.6% were admitted to ICU, and mean LOS was 12.7 days (SD: 21.5). In the primary and secondary (2015–2019, 4811 patients) cohorts, the AUROCs of the SEPSIS score for predicting in-hospital mortality were 0.63 and 0.64 respectively, which were similar to NEWS2 (0.62 and 0.67) and qSOFA (0.62 and 0.68). AUROCs for predicting ICU admission at 72 hours, and length of stay > 14 days, were similar between scores, in the primary and secondary cohorts. All scores had comparable calibration for predicting mortality.

**Conclusions:**

An EMR-based SEPSIS score shows a similar ability to predict important clinical outcomes compared with other validated scores (qSOFA and NEWS2). Because of the SEPSIS score’s simplicity, it may prove a useful tool for clinical and research applications.

## Introduction

Sepsis is a state of life-threatening multi-system organ dysfunction secondary to infection. Sepsis is a common cause for acute-care hospitalization and intensive-care unit (ICU) admission and is associated with significant morbidity and mortality [1–3]. Healthcare costs associated with acute-care hospitalization and long-term recovery from sepsis are substantial, contributing to an estimated $24 billion dollars in healthcare expenditures per year in the United States alone [4–7]. A key consideration when managing patients with sepsis is appropriate admission decision-making based on illness severity. Risk-prediction tools including the sequential organ failure assessment score (SOFA) [8], quick Sequential Organ Failure Assessment score (qSOFA) [9], and National Early Warning Score 2 (NEWS2) [10] are commonly used to approximate illness severity, guide hospital disposition decision-making, and facilitate research. These tools have shown utility in the emergency department (ED) and in assessing non-ICU admitted inpatients to detect changes in clinical status and the need for ICU transfer [11–13]. A limitation to the applicability of these tools is they rely on bedside clinical data [14–16] which pose challenges for simplicity and adaptability to clinical decision-support tools that rely on readily accessible electronic medical record (EMR) data. For instance, vital signs, mental status, and other clinical and laboratory data may not be available for all patients, may have poor inter-rater reliability, or may not be routinely evaluated [17]. These limitations may also have impacts on the generalizability across patient groups and centers.

Clinical decision-support tools are needed to prognosticate and guide clinical decision-making without the need for extensive clinical data beyond what is contained in a typical clinical evaluation for sepsis. These tools must be simple, adaptable and generalizable with similar operating characteristics to the qSOFA and NEWS2 [13, 18, 19]. Moreover, creation of a simple EMR-based predictor score may be of utility to stratify patient populations based on illness severity across jurisdictions and to allow for inter-hospital standardization and comparisons in performance. This is useful both for health-related quality of care and performance measures, as well as to facilitate large multicenter research efforts that span different forms of medical record keeping.

To address limitations of existing risk scores in sepsis, we sought to develop and validate a Simple EMR-based Predictors of Illness Severity in Sepsis (SEPSIS) Score to predict adverse outcomes, using common EMR-based laboratory data from 4 large Canadian academic hospitals. We also compared this novel predictor score to established clinical risk-prediction tools including the qSOFA and NEWS2.

## Materials and methods

### Study design

We conducted a retrospective cohort study evaluating a new SEPSIS score to predict adverse outcomes for patients admitted with suspected sepsis to four large academic teaching hospitals in the Greater Toronto Area (GTA), Canada between April 1, 2010 to March 31, 2019. The SEPSIS Score was first evaluated in a primary 2010–2015 cohort due to data availability and chart extraction and then subsequently evaluated in a secondary 2015–2019 cohort.

Our study was approved by the Research Ethics Boards (REB) of the University Health Network, Sunnybrook Health Sciences Centre, and Unity Health Toronto. This cohort study is covered by “The General Medicine Inpatient Retrospective Cohort Sub-study,” (Study ID: 15–087) approved from July 19, 2022 –July 19, 2023. Given the nature of the healthcare administrative data, participants were not required to provide informed consent. Consent as such was waived by the ethics committee. Authors did not have access to any information that could identify individual participants during or after data collection. The approval number for the REB at Unity Health Toronto is REB#: 16–148 and CTO ID#: 1392. The REB letters can be found within the uploaded supplementary materials. Unity Health Toronto Research Ethics Board is qualified through the CTO REB Qualification Program and is registered with the U.S. Department of Health and Human Services (DHHS) Office for Human Research Protection (OHRP). All procedures are in accordance with the ethical standards of the responsible committee on human experimentation (institutional or regional) and with the Helsinki Declaration of 1975.

### Data sources and study populations

We used data from hospitals participating in the GEMINI hospital research collaborative [20–28]. The primary study cohort was composed of patients admitted to or discharged from the internal medicine (GIM) services at four large Toronto academic teaching hospitals: Unity Health Toronto (St. Michael’s Hospital), University Health Network (Toronto General Hospital and Toronto Western Hospital), and Sunnybrook Health Sciences Centre. The GEMINI retrospective cohort data was accessed and analyzed between October 2021 to August 2023 for all administrative data.

### Cohort definition and eligibility

We first considered all patients admitted to or discharged from the GIM service at our study hospitals from April 1, 2010 to March 31, 2015. We included patients admitted through the ED and thus excluded patients with the following criteria: (1) planned or elective hospitalization; (2) inter-hospital transfer; or (3) age < 16. In order to capture patients with suspected sepsis, which is not necessarily accurately reflected in initial ICD-diagnostic coding given the challenge of early identification of sepsis, patients were included if they received antibiotics in at least 2 of the first 3 days after admission (or less if death occurred within that time frame) and had a positive bacterial culture within 48 hours of hospital admission, excluding screening MRSA, VRE and CPE cultures. From this group of patients, we then consecutively selected patients for retrospective chart review to extract clinical characteristics at each site (Table 1). Patients were followed from the date of admission to the earliest of death or hospital discharge.

**Table 1:** Characteristics and outcomes stratified by SEPSIS score for the primary cohort.

Overall	N = 1890	0 (n = 902)	1 (n = 705)	2 (n = 224)	3 (n = 59)
**Demographics**					
Age (median (IQR))	72 (56, 83)	71 (53, 83)	74 (59, 83)	70 (56, 84)	58 (51, 71.5)
Sex = F (%)	912 (48.3)	487 (54.0)	326 (46.2)	86 (38.4)	13 (22.0)
**Comorbidities**					
Charlson Comorbidity Index (mean (SD))	1.73 (1.86)	1.39 (1.70)	1.92 (1.92)	2.22 (2.02)	2.76 (1.91)
**Visit Characteristics**					
GIM Admission (%)	1666 (88.1)	828 (91.8)	629 (89.2)	167 (74.6)	42 (71.2)
Antibiotics within 24 hours of admission (%)	1604 (84.9)	771 (85.5)	582 (82.6)	202 (90.2)	49 (83.1)
Antibiotics from 24–48 hours of admission (%)	860 (45.5)	394 (43.7)	326 (46.2)	113 (50.4)	27 (45.8)
**Prior Medical History**					
ICU Admission in past 3 months (%)	50 (2.7)	22 (.5)	17 (2.4)	N<6	N<6
Hospitalization in last 3 months (%)	422 (22.6)	191 (21.5)	163 (23.4)	53 (24.0)	15 (25.4)
**Laboratory Features**					
Bilirubin at admit (median (IQR))	12 (7, 20)	10 (7, 15)	12 (7, 18)	16 (9.5, 31.5)	51 (39, 89.5)
Creatinine at admit (median (IQR))	93.5 (69, 140)	75 (62, 94)	131 (82.8, 195)	166 (128, 239)	148 (115, 260)
Platelet count at admit (median (IQR))	232 (172, 317)	248 (190.5, 335)	227 (172, 305.3)	199 (116, 273)	77 (55.8, 104.5)
Lactate at admit (median (IQR))	1.80 (1.30, 2.70)	1.40 (1.10, 1.70)	2.00 (1.40, 2.80)	2.80 (2.20, 4.10)	3.10 (2.50, 4.90)
**Vitals on Admission (From Chart Review)**					
Systolic Blood Pressure (mean (SD))	109.27 (22.38)	111.80 (20.56)	109.68 (23.48)	101.18 (23.95)	95.93 (17.70)
Diastolic Blood Pressure (mean (SD))	58.15 (12.75)	59.73 (11.90)	57.71 (13.41)	54.63 (12.97)	52.27 (11.74)
Abnormal Mental Status (GCS < 15) (%)	678 (35.9)	279 (30.9)	264 (37.4)	105 (46.9)	30 (50.8)
Respiratory Rate (mean (SD))	22.96 (7.15)	22.34 (6.05)	22.81 (7.50)	25.03 (8.25)	26.23 (10.94)
Heart Rate (mean (SD))	103.49 (24.38)	101.99 (22.86)	102.15 (24.64)	110.12 (25.80)	117.75 (29.71)
Mechanical Ventilation (%)	88 (4.7)	38 (4.2)	28 (4.0)	N<6	N<6
Lowest Oxygen Saturation (mean (SD))	92.68 (6.92)	92.80 (6.39)	92.63 (7.41)	92.99 (4.61)	90.40 (13.12)
Highest Temperature (mean (SD))	37.37 (1.08)	37.37 (1.05)	37.35 (1.10)	37.47 (1.12)	37.24 (1.12)
qSOFA Score (mean (SD))	1.15 (0.95)	1.00 (0.87)	1.15 (0.97)	1.61 (0.98)	1.71 (0.97)
NEWS Score (mean (SD))	6.08 (3.72)	5.57 (3.47)	6.02 (3.82)	7.71 (3.67)	8.41 (3.91)
**Positive culture within 48hrs**					
Urine (%)	968 (51.2)	470 (52.1)	375 (53.2)	100 (44.6)	23 (39.0)
Respiratory (%)	226 (12.0)	114 (12.6)	75 (10.6)	28 (12.5)	9 (15.3)
Blood (%)	309 (16.3)	108 (12.0)	129 (18.3)	53 (23.7)	19 (32.2)
Others (%)	563 (29.8)	284 (31.5)	193 (27.4)	71 (31.7)	15 (25.4)
**Outcomes**					
In Hospital Death (%)	170 (9.0)	49 (5.4)	70 (9.9)	35 (15.6)	16 (27.1)
ICU Admission (%)	351 (18.6)	114 (12.6)	131 (18.6)	80 (35.7)	26 (44.1)
ICU at 72 hours (%)	243 (12.9)	69 (7.6)	89 (12.6)	64 (28.6)	21 (35.6)
Length of Stay (mean (SD))	12.74 (21.53)	11.12 (18.47)	13.32 (24.13)	16.41 (24.72)	16.55 (16.56)

*To reduce the privacy risk that could lead to re-identification of individuals and residual disclosure of information, data on fewer than 6 admissions are suppressed. When the number of admissions can be mathematically determined from other categories, the category with the second lowest number of admissions is also suppressed.

We repeated our analyses in a recent and temporally distinct (non-overlapping) secondary cohort of hospital admissions at one study hospital from April 1 2015 to December 31 2019 (n = 4,811 patients). A single hospital (Unity Health Toronto) was used as it had integrated EMR variables that allowed calculation of NEWS2 and qSOFA scores without the need for chart review. This secondary cohort involved patients receiving systemic empiric antibiotics for a presumed infection. We did not restrict this group to those with positive cultures, which allowed us to test the performance of the score in early clinical time points where culture data is typically not available. In this secondary cohort, vital signs were available within the clinical database.

### Chart review

For the primary cohort and time period, patient vital signs were not recorded electronically at all participating hospitals, thus manual chart review of the paper or electronic medical records from the ED portion of the hospital admission for each identified patient was performed in order to obtain the necessary components of the qSOFA and NEWS2 prediction scores. See Supplemental Methods in S1 File for further details.

### Selection of SEPSIS score predictors

We *a priori* chose predictors to include in the SEPSIS score based on prior evidence [4, 12, 29], generalizability (e.g. use in health care systems without access to a variety of vitals/mental status data), simplicity (a straightforward additive score), and adaptability (for clinical and research applications). We did not select parameters using statistical approaches, which naturally may come at the cost of some test performance. We also chose to create the score without using changes from baseline values, rather using only the values available (or not) at the time of admission from the ED. We chose this approach as patients often did not have prior baseline values available, or the previous laboratory values in the electronic medical record did not accurately reflect the patient’s most recent baseline. Specifically, for a given lab test, the first measurement collected within 24 hours prior to and 48 hours after inpatient admission was used for score calculation. We prioritized investigations that are commonly available for patients presenting with sepsis syndromes. We selected the following variables and cut-offs based on the standardized upper limits of normal at participating institutions, as well as the literature supporting their association with poor outcomes in sepsis [4]: (1) a creatinine (Cr) of greater than 125 (μmol/L); (2) a total bilirubin (Tbili) greater than 35 (μmol/L); (3) a platelet (Plt) count less than 100 (x 10^9), and (4) a lactate (Lac) greater than 2 (mmol/L). For simplicity, we assigned each abnormal predictor (outside of the cut-off) one point for the score, with a maximum total score of four, and a minimum score of zero. If a particular value was unavailable (e.g., there was no lactate ordered), then no point was given for that result type. We felt this was justifiable as the absence of a standard blood test is likely reflective of a patient with a lower severity of illness.

### Calculation of other outcome prediction scores (NEWS2, QSOFA)

We generated a qSOFA score [9] and NEWS2 score [10] for each patient from EMR data and chart review [12]. Any missing variables were imputed as normal. See Supplemental Methods in S1 File for further details.

### Outcomes

Our primary outcome of interest was in-hospital mortality. Patients were followed from the date of admission to the earliest of death or hospital discharge. We also evaluated other relevant secondary outcomes, including: (1) ICU admission or death at 72 hours from hospital admission and (2) total length of hospital stay (in days).

#### Data analysis

**Fig 1:**
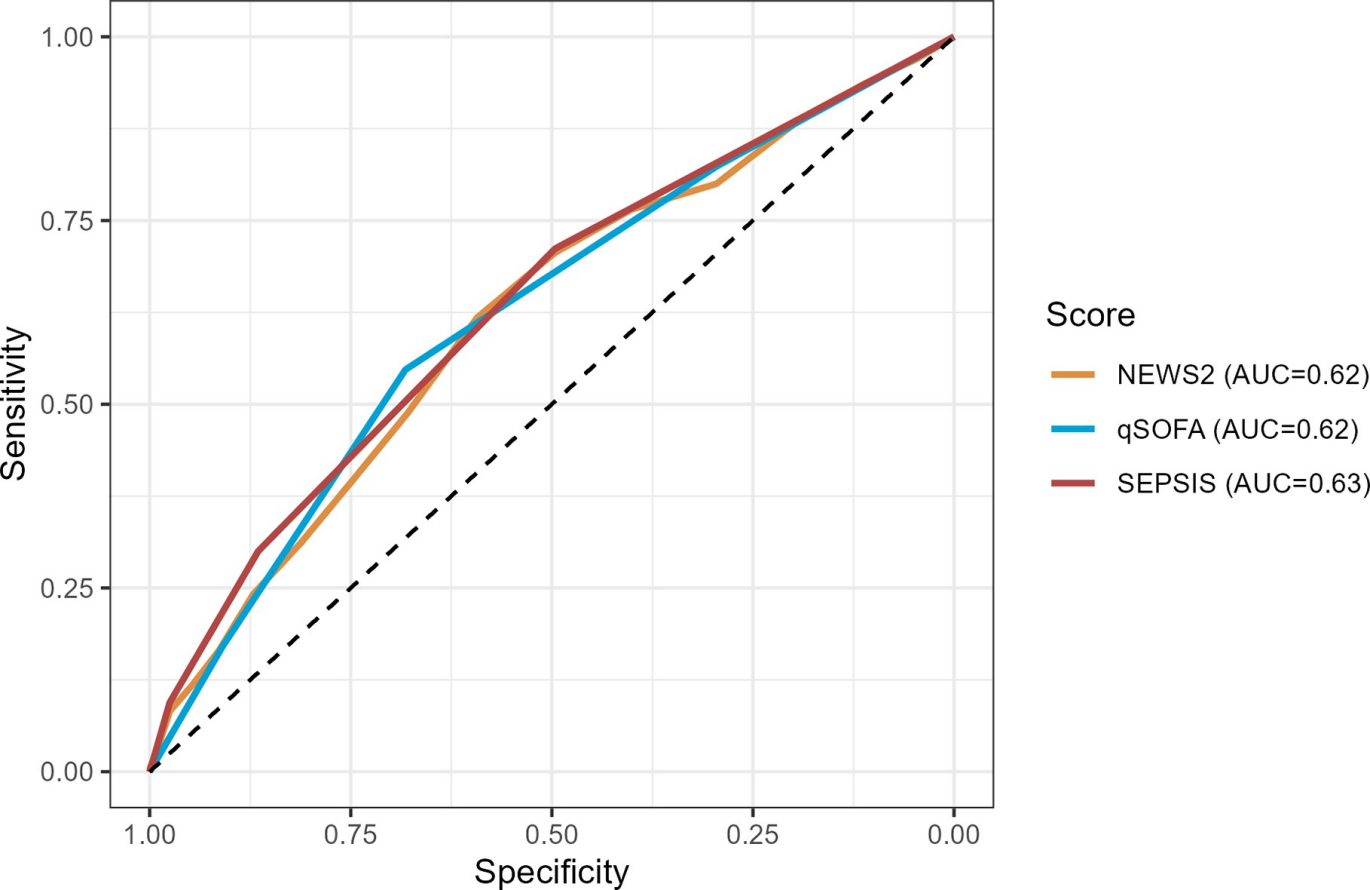
Receiver operating characteristics curves for prediction of mortality for SEPSIS score and other risk prediction scores, for the primary cohort.

**Fig 2:**
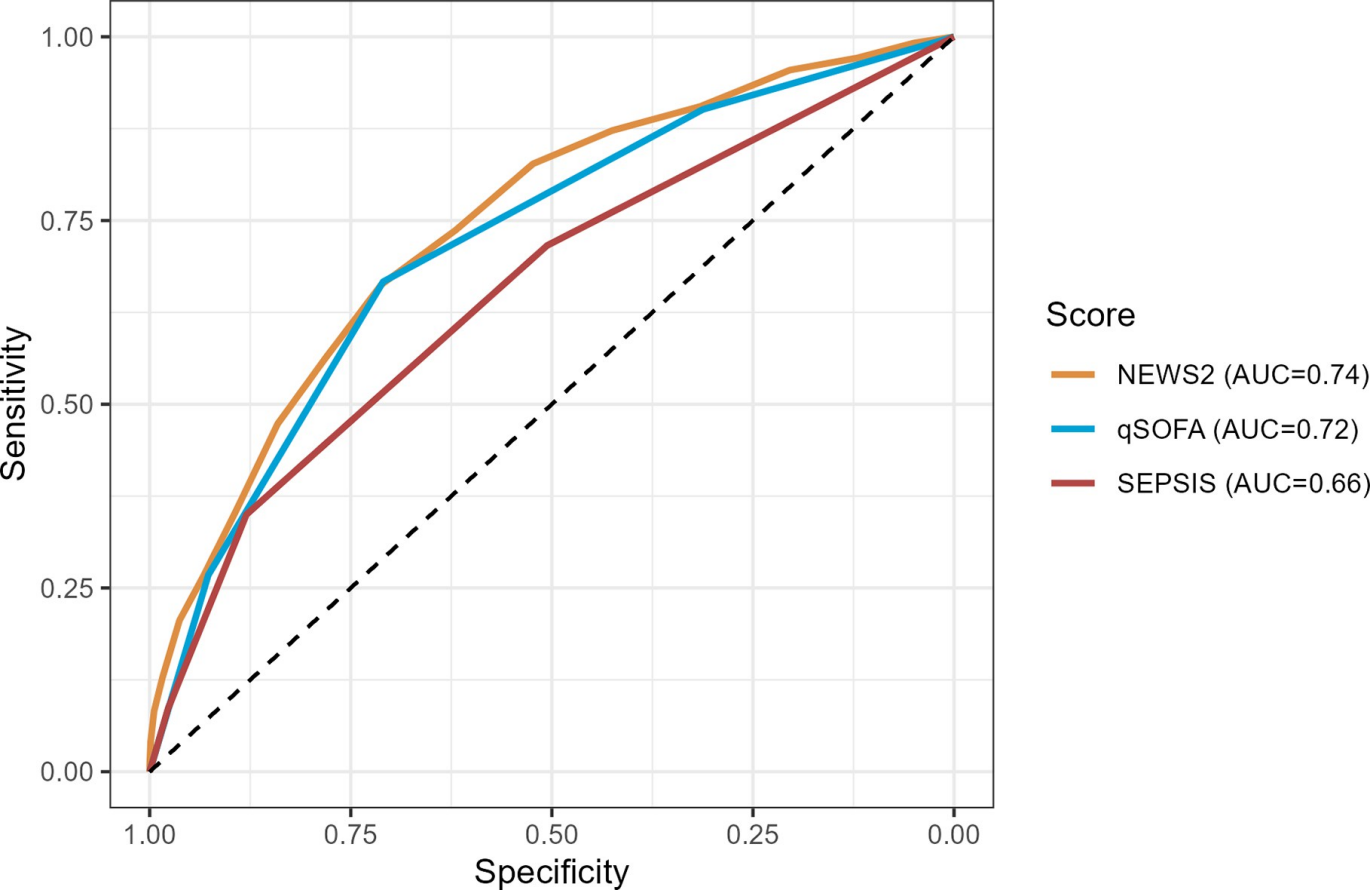
Receiver operating characteristics curves for prediction of ICU admission at 72 hrs for SEPSIS score and other risk prediction scores, for the primary cohort.

**Fig 3:**
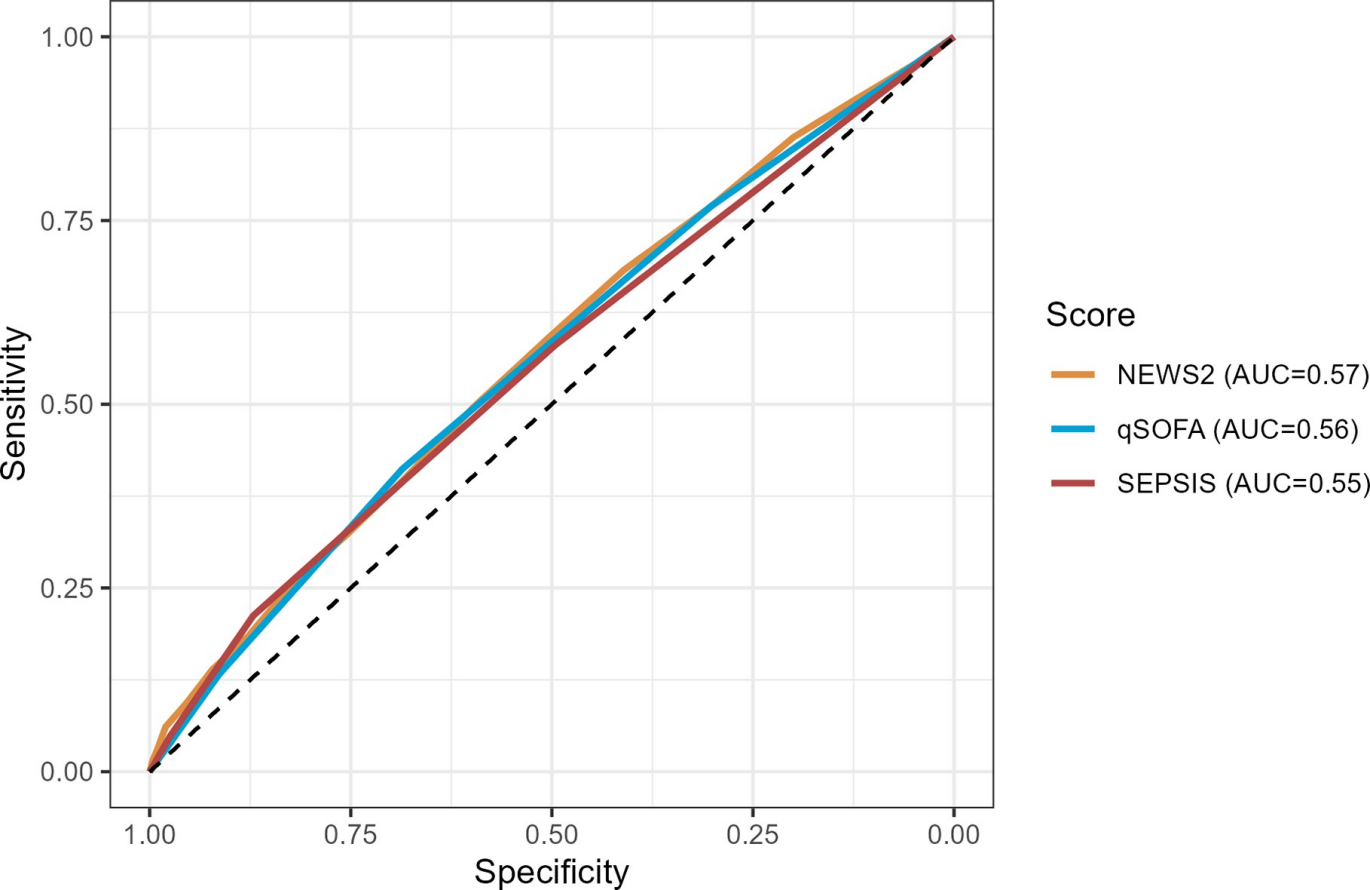
Receiver operating characteristics curves for prediction of prolonged hospital admission (LOS > 14 days) for SEPSIS score and other risk prediction scores, for the primary cohort.

To describe the level of comorbidity in our patient population, we computed the Charlson Comorbidity Index [30]. We provide data on visit characteristics (service of admission) and an estimate of recent healthcare utilization including ICU admission and GIM hospital admission within the 3 months preceding the index hospitalization to one of the GEMINI hospitals. In our SEPSIS score analysis, we collapsed those with a score of ≥ 3 into one grouping (3+) due to low patient counts. We calculated the sensitivity, specificity, negative predictive value (NPV) and positive predictive value (PPV) for mortality, ICU admission or death at 72 hours of hospitalization (or death), and length of stay using the qSOFA (1, 2, 3), NEWS2 (5, 7, 10), and SEPSIS score (1, 2, 3), compared to each score’s referent group. We selected the following cut points to reflect low, medium, and high-risk categorizations of each score: qSOFA (0–1, 2, 3), NEWS2 (0–4, 5–6, 7+), and SEPSIS (0–1, 2, 3). We plotted mortality by risk categorization (low, medium, high) to assess score calibration. Further, we computed the area under the receiving operating characteristic curve (AUROC) for each score derived from our cohort for comparisons between the qSOFA, NEWS2, and SEPSIS scores (Figs 1–3). The DeLong method implemented in the ‘pROC’ R package was used to compare AUROCs between scores [31]. An alpha level of 0.05 was used to determine statistical significance. All analyses were performed using R version 4.12 (https://www.R-project.org/).

## Results

The primary cohort included 1,890 patients admitted to a medical service at 4 academic teaching hospitals from April 1st, 2010 to March 31st, 2015. The baseline characteristics of the cohort, overall and stratified by SEPSIS predictor score, are displayed in Table 1. The median age of the cohort was 72 years (IQR: 56–83) and approximately half were female (48.3%). Pathogens isolated from clinical cultures and antibiotics received are presented in S1 and S2 Tables in S1 File. 170 (9%) of patients died during the index hospitalization, and 243 (12.9%) of the total cohort were admitted to the ICU at 72 hours from admission. The mean length of stay of the cohort was 12.7 days (SD: 21.5). Patient characteristics and outcomes changed with increasing SEPSIS score (Table 1). Adverse outcomes including in-hospital mortality, ICU admission, and length of stay increased monotonically with increasing risk predictor score with 27.1% of those in the highest risk group having in hospital mortality, 44.1% being admitted to an ICU, and with a mean length of stay of 16.6 (SD: 16.6) days.

**Fig 4:**
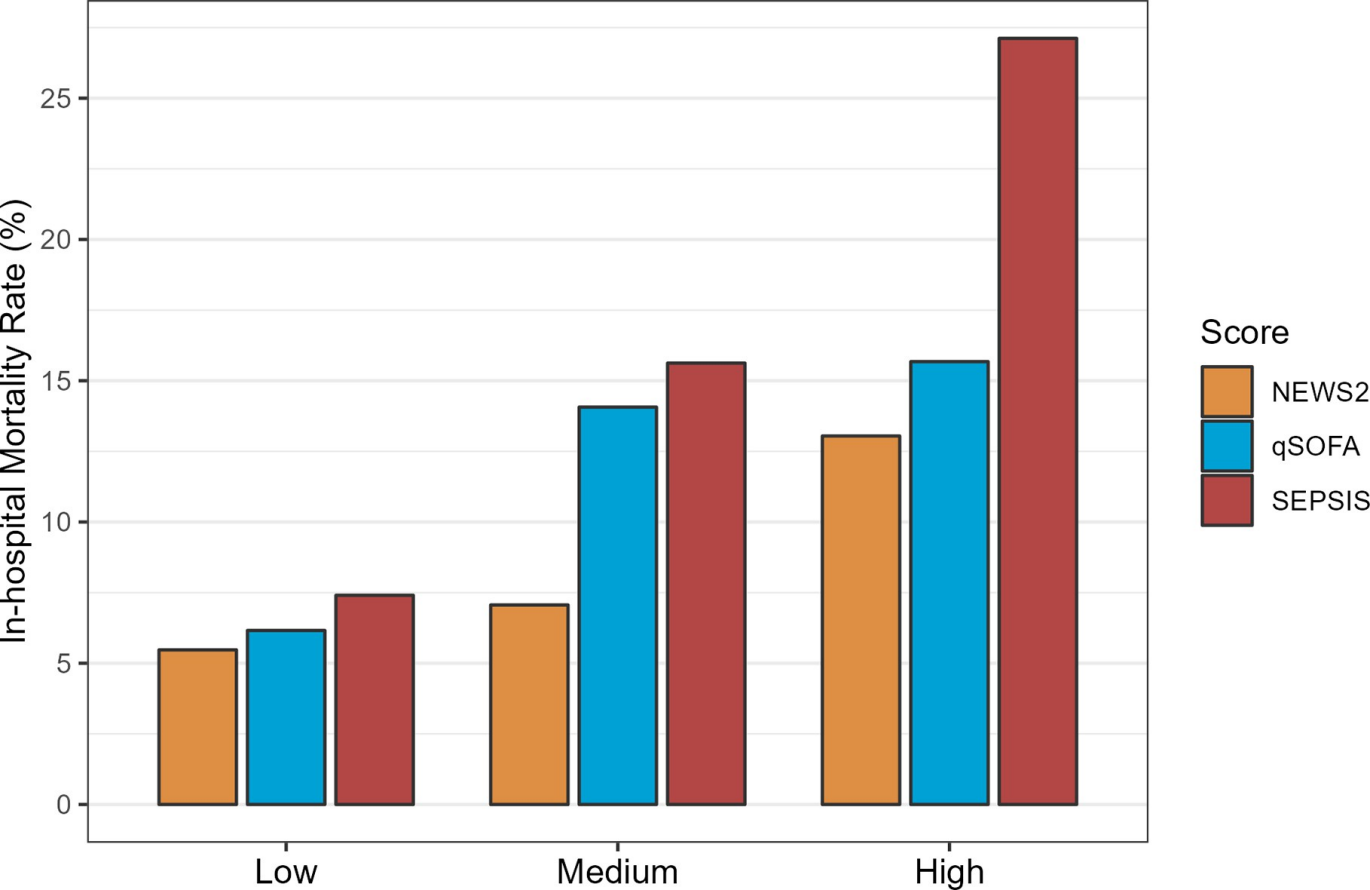
Bar graph of mortality risk by predictor score (low, medium, high) and score type, for the primary cohort.

The SEPSIS score has an AUROC for predicting mortality of 0.63 [95%CI: 0.59, 0.68], similar to the qSOFA (0.62 [95%CI: 0.58, 0.67]) and NEWS2 (0.62 [95%CI: 0.57, 0.66]) scores (Fig 1 and S3 Table in S1 File). A high SEPSIS score (3+) had high specificity (0.98) and a non-zero SEPSIS score (1+) had modest sensitivity (0.71) for predicting mortality (S4A Table in S1 File). The SEPSIS score also had similar AUROC, and test characteristics, to the qSOFA and NEWS2 scores for predicting ICU admission at 72 hours and length of stay > 14 days (Figs 2 and 3 and S4B, S4C Tables in S1 File), though the performance characteristics of all scores for predicting length of stay was poor. The negative predictive values for all the scores were generally favourable, due in part to the low overall prevalence of the outcomes in the cohort. The SEPSIS score appeared well calibrated with mortality across the range of low/medium/high scores (Fig 4) and paralleled the calibration of qSOFA and NEWS2.

We repeated our analyses in the secondary cohort (2015–2019) which specifically included all patients who received systemic antibiotic exposure (regardless of culture status) (S5 Table in S1 File) and represents a broader group of patients with suspected sepsis. Our findings were concordant with the 2010–2015 cohort, namely that the SEPSIS score had similar AUROC magnitudes compared to qSOFA and NEWS2 scores for predicting mortality, ICU admission at 72 hours, and length of stay > 14 days (S1–S3 Figs and S6 Table in S1 File). As with the 2010–2015 cohort, the SEPSIS score appeared well calibrated with mortality in this 2015–2019 cohort across the range of low/medium/high scores (S4 Fig in S1 File) and paralleled the calibration of qSOFA and NEWS2.

## Discussion

In this retrospective observational cohort study of patients admitted to medical services at 4 Canadian hospitals, we developed a novel simple electronic medical record-based predictors of illness severity in sepsis (SEPSIS) score. We found its performance for predicting adverse clinical outcomes, including mortality, was similar to several commonly used risk prediction tools, yet it only required the results of 4 simple and commonly ordered laboratory tests.

While the development of this SEPSIS score was intentionally not statistically driven, it ultimately showed comparable performance characteristics to the qSOFA and NEWS2, and with the potential for strong generalizability. Notably, AUCs were similar across all scores for the primary outcome of mortality, as well as other secondary outcomes, which supports comparable ability to discriminate these outcomes. Test characteristics, including sensitivity and specificity, vary by the specific score thresholds used, but were similar by low, moderate, and high classifications. While overall AUCs for predicting mortality were consistent across all three scores, they were relatively modest, although of similar magnitudes to the existing literature [32]. All three models appeared similarly calibrated for predicting increasing mortality with increasing predictor scores, in particular differentiating low risk of mortality from medium to high risk.

Our data have several important clinical implications. First, these data may assist in real-time decision-making to guide patient disposition (ICU vs. medical ward) and to help inform illness severity to guide goals of care discussions with families. Their use for facilitating discharge from the ED was not specifically evaluated (e.g. non-admitted patients) and would also depend upon physician risk tolerance. Because they represent commonly available pieces of information in electronic medical records, they can be easily incorporated into medical record-based tools for real-time risk stratification, even in settings where vital signs are not included in the EMR. Second, the SEPSIS score could be used to compare performance and provide standardization across hospitals in different jurisdictions, given that it is driven by only a few common variables. While many current risk prediction tools rely on advanced statistical modelling or machine-learning for their development, there is still a need for transparent stratification tools with low risk of bias that can be applied simply and easily across disparate datasets [33].

Our study has several limitations. First, we made the assumption that patients receiving antibiotics for multiple days upon admission and with positive cultures were suspected of having clinically relevant sepsis. We used this classification given the challenge of both defining and identifying early sepsis, as well as inconsistency in administrative data definitions used to identify sepsis [4, 34]. We are somewhat limited in our ability more definitively identify sepsis given that we have to infer suspicion of infection via antimicrobial prescribing and culture collection/positivity. This is a commonly used approach in other cohort studies and we believe it to be a clinically relevant cohort classification [3, 13]. Because we defined suspected sepsis as patients receiving antibiotics and having a positive culture, we have not considered the population of patients with suspected sepsis and negative cultures. However, the comparable performance of our predictor score in the secondary cohort of patients (2015 to 2019) identified based on antibiotic prescriptions alone, indicates that this score will likely be generalizable to all patients with suspected sepsis. It is also worth noting the majority of patients with confirmed infection did have an infection of urinary origin, which may impact on generalizability, though conversely it is reflective of the typical distribution of sepsis etiologies.

## Conclusion

We present a simple EMR-based SEPSIS score to predict mortality and other adverse clinical outcomes in patients with sepsis with similar performance to other more complex and less generalizable commonly used prediction scores. This SEPSIS score requires further validation in separate regions and clinical contexts, but with the promise of ease of use and generalizability for both clinical and research applications.

## Supporting information

S1 Checklist Completed strobe checklist. (DOC)

S2 Checklist STROBE statement—Checklist of items that should be included in reports of *cohort studies*. (DOCX)

S1 File Supporting materials including methods, figures, and tables. (DOCX)

S2 File Sepsis score summary. (DOCX)
