# Supplementary material for: A simple electronic medical record-based predictors of illness severity in sepsis (sepsis) score
Source: PLoS One. 2024 Jun 26;19(6):e0299473. doi: 10.1371/journal.pone.0299473 (PMC11206954; doi:10.1371/journal.pone.0299473)

**SUPPLEMENTAL MATERIALS**

**Supplemental Methods**

*Data Sources and Study Populations*

GTA is home to more than six million people and is the most populous metropolitan region in Canada. All participating hospitals are publicly-funded, operate independently and provide tertiary and/or quaternary care. GEMINI collects clinical and administrative data extracted from hospital computer systems, with 98-100% accuracy across key data types.(25,26) The GEMINI dataset has previously been described in detail with several studies examining healthcare utilization and healthcare outcomes, as well as a range of infectious disease topics.(21–29)

*Chart Review*

Patient vital signs were not recorded electronically at all participating hospitals. In particular, vital signs recorded in the ED were commonly documented on paper charts. Thus, manual chart review of the paper or electronic medical records from the ED portion of the hospital admission for each identified patient was performed by AM, AK, IFAJ, MB, and RK. We collected the following variables during the ED stay: highest or lowest systolic and diastolic blood pressure, abnormal Glasgow Coma Scale (GCS) of less than 15 or a documented abnormal mental status, highest and lowest respiratory rate, highest and lowest heart rate, lowest oxygen saturation, and highest temperature. We used these data to compute the qSOFA score and NEWS2 score. These data were abstracted using REDCAP (V6.18.1). Antibiotic prescribing was extracted from the EMR at each site using the GEMINI data set and classified into common classes of antibiotics (Supplementary Table 1). Microbial culture data was similarly extracted from the GEMINI Data set and mapped using common bacterial taxonomies (Supplementary Table 2). We assessed interobserver variability via parallel chart review for 339 (8%) patients, with two separate abstracters for each chart. When assessing interobserver variability for chart abstraction of ED vitals signs, we found that for continuous variables that were present in more than 10% of reviewed charts, there was strong correlations ranging from 0.85-0.99, and for binary/categorical variables we found Cohen’s kappa values ranging from 0.79 - 0.93.

*Calculation of Other Outcome Prediction Scores (NEWS2, QSOFA)*

We generated a qSOFA score(10) and NEWS2 score(11) for each patient from EMR data and chart review. We made the following interpretations when assigning the qSOFA score: (1) if mechanically ventilated, we assumed a respiratory rate of > 22 breaths per minute. We similarly generated a NEWS2 score[^11^](https://paperpile.com/c/Nxe1tL/hu6c) for each patient from the EMR and chart review. The following interpretations were used to compute the modified NEWS2 score: (1) if the patient was mechanically ventilated, the respiratory rate was assumed to be ≥ 25, (2) level of consciousness was dichotomized to either alert (normal) or abnormal defined as GCS < 15 or an abnormal mental status was documented in the chart abstraction, (3) the oxygen saturation was scored using Scale 1 only and Scale 2, which is dedicated for use in patients with hypercapnic respiratory failure who have a clinically recommended oxygen saturation between 88 to 92% was ignored, and (4) air or oxygen score was not included. Any missing variables were imputed as normal. In the secondary cohort, positive Confusion Assessment Method (CAM) was used as a proxy for GCS < 15, as GCS data is not systematically recorded.

**Supplemental Tables**

**Supplemental Table 1. Prevalence of antibiotic use by class (%), for the primary cohort.**

| **Group** | **Overall** |
| --- | --- |
| n | 4269 |
| 1st Generation Cephalosporins | 186 (4.4) |
| 2nd Generation Cephalosporins | 76 (1.8) |
| 3rd Generation Cephalosporins | 670 (15.7) |
| 4th Generation Cephalosporins | N<6 |
| Aminoglycosides | 76 (1.8) |
| Carbapenems | 213 (5) |
| Fluoroquinolones | 916 (21.5) |
| Fosfomycins | N<6 |
| Furans | 89 (2.1) |
| Glycopeptide | 231 (5.4) |
| Lincosamides | 134 (3.1) |
| Lipopeptide | N<6 |
| Macrolides | 488 (11.4) |
| Methylimidazole | 337 (7.9) |
| Oxazalidinone | N<6 |
| Penicillins | 625 (14.6) |
| Sulfa | 153 (3.6) |
| Tetracyclines | 61 (1.4) |

**Supplemental Table 2. Microbiology of positive cultures by bacterial genus, for the primary cohort.**

|  | **Overall** |
| --- | --- |
| n | 2420 |
| *Acinetobacter species* | N<6 |
| *Actinomyces species* | N<6 |
| *Aerococcus species* | N<6 |
| *Bacillus species* | 13 (0.5) |
| *Bacteroides fragilis* | N<6 |
| *Citrobacter species* | 13 (0.5) |
| *Corynebacterium species* | N<6 |
| *Enterobacter species* | 28 (1.2) |
| *Enterococcus species* | 288 (11.9) |
| *Escherichia coli* | 629 (26) |
| *Fusobacterium species* | N<6 |
| *Haemophilus influenzae* | 44 (1.8) |
| *Klebsiella species* | 146 (6) |
| *Morganella species* | 25 (1) |
| *Others Bacteria* | 84 (3.5) |
| *Proteus species* | 70 (2.9) |
| *Pseudomonas species* | 151 (6.2) |
| *Serratia species* | 16 (0.7) |
| *Staphylococcus species* | 656 (27.1) |
| *Stenotrophomonas maltophilia* | 14 (0.6) |
| *Streptococci species* | 228 (9.4) |

^*^ To reduce the privacy risk that could lead to re-identification of individuals and residual disclosure of information, data on fewer than 6 admissions are suppressed. When the number of admissions can be mathematically determined from other categories, the category with the second lowest number of admissions is also suppressed.

***Primary Cohort (2010-2015)***

**Supplemental Table 3. AUROC of mortality prediction by score and their pairwise comparisons for the primary cohort.**

| **Score** | **AUC (95% CI)** | **Difference in AUC (95% CI) versus SEPSIS** | **Difference in AUC (95% CI) versus qSOFA** |
| --- | --- | --- | --- |
| **SEPSIS** | 0.63 (0.59, 0.68) | - | - |
| **qSOFA** | 0.62 (0.58, 0.67) | -0.01 (-0.07, 0.05) | - |
| **NEWS2** | 0.62 (0.57, 0.66) | -0.01 (-0.07, 0.04) | 0.00 (-0.03, 0.02) |

**Supplemental Table 4a. Test characteristics of SEPSIS Score for mortality compared to other validated risk prediction scores.**

|  | **Sensitivity** | **Specificity** | **PPV** | **NPV** |
| --- | --- | --- | --- | --- |
| **qSOFA_1+** | 0.82 (0.76, 0.88) | 0.3 (0.27, 0.32) | 0.1 (0.09, 0.12) | 0.94 (0.92, 0.96) |
| **qSOFA_2+** | 0.55 (0.47, 0.62) | 0.68 (0.66, 0.7) | 0.15 (0.12, 0.18) | 0.94 (0.92, 0.95) |
| **qSOFA_3+** | 0.17 (0.12, 0.24) | 0.91 (0.89, 0.92) | 0.16 (0.11, 0.22) | 0.92 (0.9, 0.93) |
| **NEWS_5+** | 0.76 (0.69, 0.83) | 0.4 (0.38, 0.43) | 0.11 (0.09, 0.13) | 0.95 (0.93, 0.96) |
| **NEWS_7+** | 0.62 (0.54, 0.69) | 0.59 (0.57, 0.62) | 0.13 (0.11, 0.16) | 0.94 (0.92, 0.95) |
| **NEWS_10+** | 0.31 (0.24, 0.39) | 0.81 (0.79, 0.83) | 0.14 (0.11, 0.18) | 0.92 (0.91, 0.94) |
| **SEPSIS_1+** | 0.71 (0.64, 0.78) | 0.5 (0.47, 0.52) | 0.12 (0.1, 0.14) | 0.95 (0.93, 0.96) |
| **SEPSIS_2+** | 0.3 (0.23, 0.37) | 0.87 (0.85, 0.88) | 0.18 (0.14, 0.23) | 0.93 (0.91, 0.94) |
| **SEPSIS_3+** | 0.09 (0.05, 0.15) | 0.98 (0.97, 0.98) | 0.27 (0.16, 0.4) | 0.92 (0.9, 0.93) |

**Supplemental Table 4b. Test characteristics of SEPSIS Score for ICU at 72 hours compared to other validated risk prediction scores.**

|  | **Sensitivity** | **Specificity** | **PPV** | **NPV** |
| --- | --- | --- | --- | --- |
| **qSOFA_1+** | 0.9 (0.86, 0.94) | 0.31 (0.29, 0.34) | 0.16 (0.14, 0.18) | 0.96 (0.93, 0.97) |
| **qSOFA_2+** | 0.67 (0.6, 0.73) | 0.71 (0.69, 0.73) | 0.25 (0.22, 0.29) | 0.94 (0.92, 0.95) |
| **qSOFA_3+** | 0.27 (0.21, 0.33) | 0.93 (0.91, 0.94) | 0.35 (0.28, 0.42) | 0.9 (0.88, 0.91) |
| **NEWS_5+** | 0.87 (0.82, 0.91) | 0.43 (0.4, 0.45) | 0.18 (0.16, 0.21) | 0.96 (0.94, 0.97) |
| **NEWS_7+** | 0.74 (0.68, 0.79) | 0.62 (0.6, 0.64) | 0.22 (0.19, 0.25) | 0.94 (0.93, 0.95) |
| **NEWS_10+** | 0.47 (0.41, 0.54) | 0.84 (0.82, 0.86) | 0.31 (0.26, 0.35) | 0.92 (0.9, 0.93) |
| **SEPSIS_1+** | 0.72 (0.65, 0.77) | 0.51 (0.48, 0.53) | 0.18 (0.15, 0.2) | 0.92 (0.9, 0.94) |
| **SEPSIS_2+** | 0.35 (0.29, 0.41) | 0.88 (0.86, 0.9) | 0.3 (0.25, 0.36) | 0.9 (0.89, 0.92) |
| **SEPSIS_3+** | 0.09 (0.05, 0.13) | 0.98 (0.97, 0.98) | 0.36 (0.24, 0.49) | 0.88 (0.86, 0.89) |

**Supplemental Table 4c. Test characteristics of SEPSIS Score for LOS > 14 days compared to other validated risk prediction scores.**

|  | **Sensitivity** | **Specificity** | **PPV** | **NPV** |
| --- | --- | --- | --- | --- |
| **qSOFA_1+** | 0.77 (0.73, 0.81) | 0.3 (0.28, 0.33) | 0.27 (0.25, 0.3) | 0.8 (0.76, 0.83) |
| **qSOFA_2+** | 0.41 (0.37, 0.46) | 0.69 (0.66, 0.71) | 0.31 (0.27, 0.34) | 0.78 (0.75, 0.8) |
| **qSOFA_3+** | 0.13 (0.1, 0.17) | 0.91 (0.9, 0.93) | 0.34 (0.27, 0.41) | 0.76 (0.74, 0.78) |
| **NEWS_5+** | 0.68 (0.64, 0.72) | 0.41 (0.38, 0.44) | 0.28 (0.25, 0.31) | 0.79 (0.76, 0.82) |
| **NEWS_7+** | 0.5 (0.45, 0.54) | 0.6 (0.57, 0.62) | 0.29 (0.26, 0.33) | 0.78 (0.75, 0.8) |
| **NEWS_10+** | 0.26 (0.22, 0.3) | 0.82 (0.8, 0.84) | 0.33 (0.28, 0.38) | 0.77 (0.74, 0.79) |
| **SEPSIS_1+** | 0.58 (0.53, 0.62) | 0.5 (0.47, 0.52) | 0.28 (0.25, 0.31) | 0.78 (0.75, 0.8) |
| **SEPSIS_2+** | 0.21 (0.18, 0.25) | 0.87 (0.85, 0.89) | 0.36 (0.3, 0.42) | 0.77 (0.75, 0.79) |
| **SEPSIS_3+** | 0.05 (0.03, 0.07) | 0.97 (0.96, 0.98) | 0.39 (0.27, 0.53) | 0.75 (0.73, 0.77) |

***Secondary Cohort (2015-2019)***

**Supplemental Table 5. Characteristics and Outcomes Stratified by SEPSIS Score.**

| **Overall** | **N** | **0** | **1** | **2** | **3** |
| --- | --- | --- | --- | --- | --- |
| n | 4811 | 2360 | 1711 | 612 | 128 |
| **Demographics** |  |  |  |  |  |
| Age (median (IQR)) | 65.00 (52.00, 78.00) | 62.00 (49.00, 77.00) | 67.00 (55.00, 78.00) | 68.00 (56.00, 79.00) | 61.00 (52.00, 71.00) |
| Gender = F (%) | 1909 (39.7) | 1050 (44.5) | 649 (37.9) | 173 (28.3) | 37 (28.9) |
| **Comorbidities** |  |  |  |  |  |
| Charlson Comorbidity Index (mean (SD)) | 1.34 (1.65) | 1.10 (1.51) | 1.45 (1.67) | 1.73 (1.79) | 2.36 (2.08) |
| **Visit Characteristics** |  |  |  |  |  |
| GIM Admission (%) | 4374 (90.9) | 2243 (95.0) | 1550 (90.6) | 488 (79.7) | 93 (72.7) |
| Antibiotics within 24 hours of admission (%) | 4403 (91.5) | 2153 (91.2) | 1569 (91.7) | 565 (92.3) | 116 (90.6) |
| Antibiotics from 24-48 hours of admission (%) | 2024 (42.1) | 911 (38.6) | 749 (43.8) | 290 (47.4) | 74 (57.8) |
| **Prior Medical History** |  |  |  |  |  |
| ICU Admission in past 3 months (%) | 318 (6.7) | 147 (6.3) | 108 (6.4) | 54 (9.0) | 9 (7.2) |
| Hospitalization in last 3 months (%) | 1424 (30.1) | 684 (29.5) | 511 (30.4) | 192 (31.8) | 37 (29.6) |
| **Laboratory Features** |  |  |  |  |  |
| Bilirubin at admit (median (IQR)) | 11 (8, 18) | 10 (7, 15) | 11 (8, 17) | 16 (9, 28) | 46.5 (36, 73.25) |
| Creatinine at admit (median (IQR)) | 87 (65, 132) | 72 (58, 89) | 109 (73, 180) | 158 (123.5, 253) | 162 (126.5, 262) |
| Platelet count at admit (median (IQR)) | 237 (175, 311) | 254 (197.25, 325.75) | 230 (172, 300) | 209 (134, 289.5) | 87 (58, 152) |
| Lactate at admit (median (IQR)) | 1.80 (1.30, 2.50) | 1.40 (1.10, 1.70) | 2.10 (1.50, 2.80) | 2.60 (2.10, 3.40) | 2.80 (2.30, 4.40) |
| **Vitals on Admission (From Database)** |  |  |  |  |  |
| Lowest Systolic Blood Pressure (mean (SD)) | 127.43 (26.30) | 130.91 (24.58) | 127.27 (26.59) | 116.72 (28.45) | 115.89 (25.62) |
| Lowest Diastolic Blood Pressure (mean (SD)) | 74.66 (14.99) | 76.86 (14.09) | 73.90 (15.07) | 69.20 (16.41) | 69.98 (14.69) |
| Abnormal Mental Status (positive CAM) (%) | 924 (21.5) | 385 (18.9) | 351 (22.2) | 149 (26.6) | 39 (32.2) |
| Highest Respiratory Rate (mean (SD)) | 21.92 (31.14) | 21.25 (7.44) | 21.48 (6.81) | 25.83 (86.01) | 21.67 (6.31) |
| Highest Heart Rate (mean (SD)) | 100.74 (22.72) | 99.14 (20.29) | 101.51 (24.59) | 103.57 (25.22) | 106.70 (24.10) |
| Mechanical Ventilation (%) | 66 (1.6) | 19 (1.0) | 27 (1.9) | N<6 | N<6 |
| Lowest Oxygen Saturation (mean (SD)) | 95.59 (5.15) | 95.63 (5.07) | 95.69 (4.45) | 95.06 (6.73) | 95.94 (6.51) |
| Highest Temperature (mean (SD)) | 37.15 (1.29) | 37.11 (1.20) | 37.19 (1.31) | 37.15 (1.49) | 37.20 (1.69) |
| qSOFA Score (mean (SD)) | 0.66 (0.70) | 0.56 (0.62) | 0.69 (0.74) | 0.92 (0.76) | 0.90 (0.77) |
| NEWS Score (mean (SD)) | 4.18 (2.80) | 3.77 (2.59) | 4.31 (2.89) | 5.19 (2.90) | 5.34 (2.99) |
| **Outcomes** |  |  |  |  |  |
| In Hospital Death (%) | 268 ( 5.6) | 78 ( 3.3) | 98 ( 5.7) | 76 (12.4) | 16 (12.5) |
| ICU Admission (%) | 1021 (21.2) | 332 (14.1) | 395 (23.1) | 229 (37.4) | 65 (50.8) |
| ICU at 72 hours (%) | 595 (12.4) | 157 ( 6.7) | 237 (13.9) | 151 (24.7) | 50 (39.1) |
| Length of Stay (median (IQR)) | 5.63 (3.02, 10.70) | 4.80 (2.68, 9.15) | 5.88 (3.42, 11.02) | 7.52 (3.92, 15.54) | 10.57 (5.61, 19.54) |

* To reduce the privacy risk that could lead to re-identification of individuals and residual disclosure of information, data on fewer than 6 admissions are suppressed. When the number of admissions can be mathematically determined from other categories, the category with the second lowest number of admissions is also suppressed.

**Supplemental Table 6. AUROC of mortality prediction by score and their pairwise comparisons for the secondary cohort.**

| **Score** | **AUC (95% CI)** | **Difference in AUC (95% CI) versus SEPSIS** | **Difference in AUC (95% CI) versus qSOFA** |
| --- | --- | --- | --- |
| **SEPSIS** | 0.64 (0.61, 0.67) | - | - |
| **qSOFA** | 0.68 (0.66, 0.71) | 0.04 (0.00, 0.09) | - |
| **NEWS2** | 0.67 (0.64, 0.70) | 0.03 (-0.02, 0.07) | -0.02 (-0.04, 0.00) |

**Supplemental Figures**

***Secondary Cohort (2015-2019)***

**Supplemental Figure 1. Receiver operating characteristics curves for prediction of mortality for SEPSIS score and other risk prediction scores.**


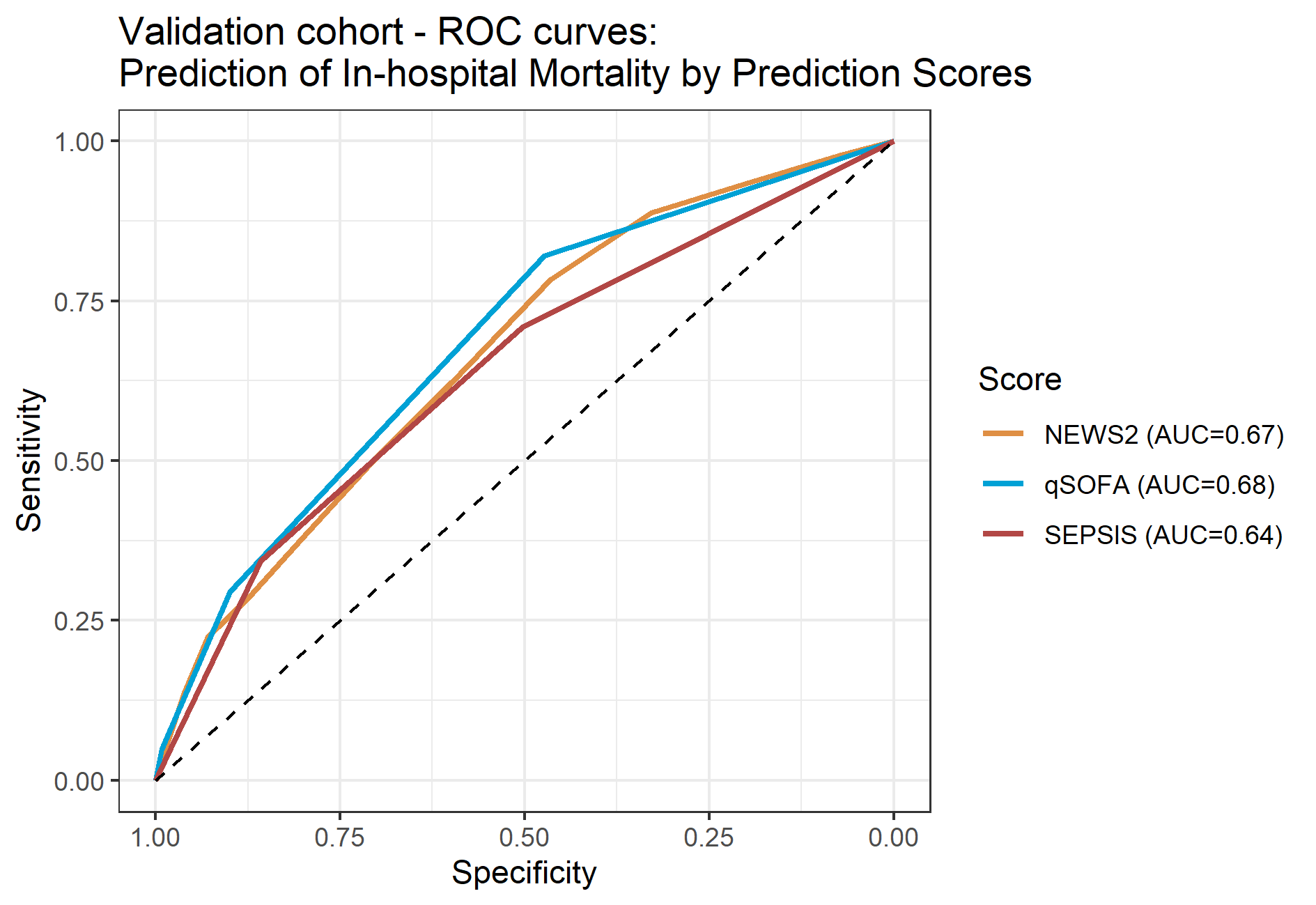


**Supplemental Figure 2. Receiver operating characteristics curves for prediction of ICU admission at 72**

**hrs for SEPSIS score and other risk prediction scores.**


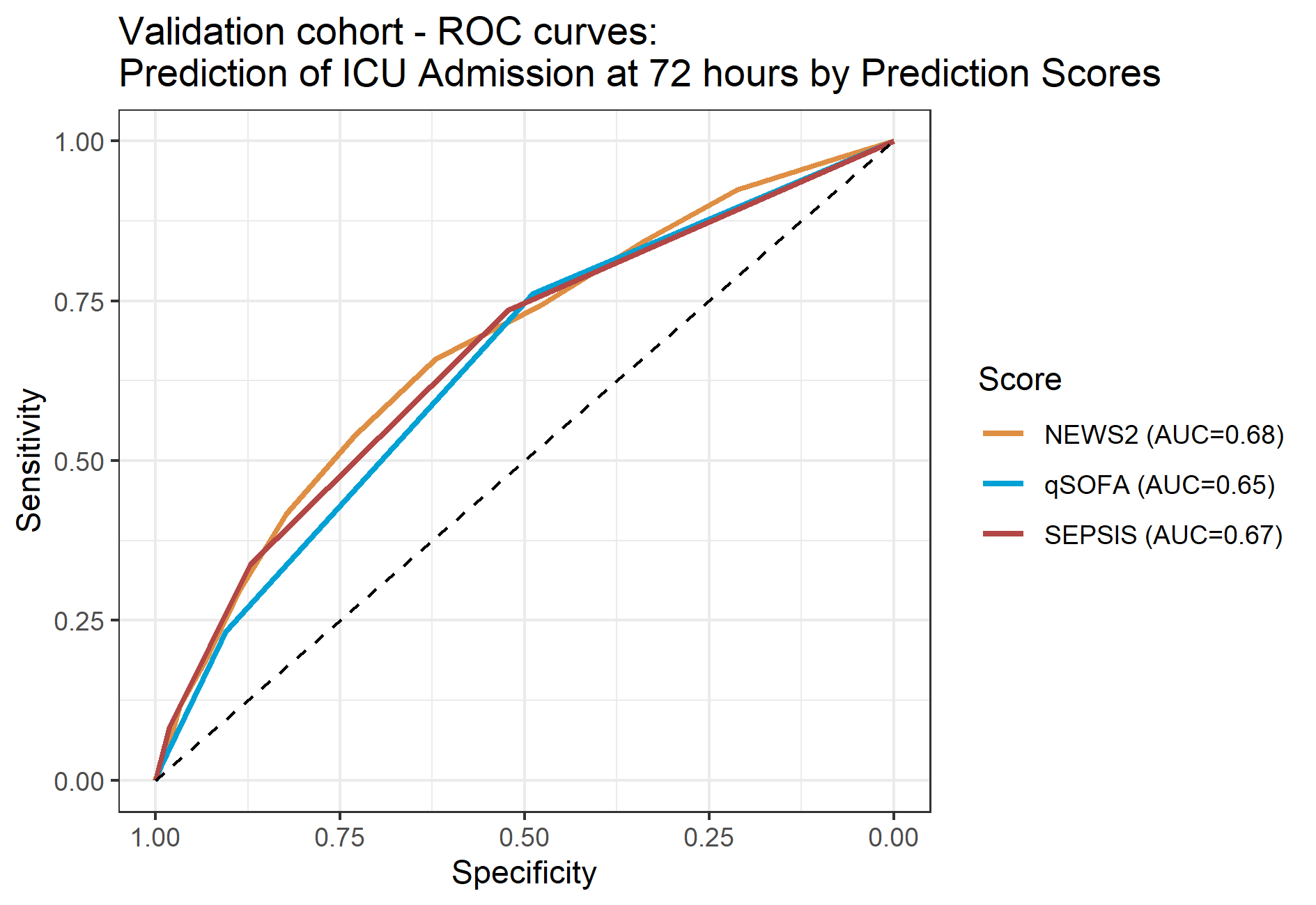


**Supplemental Figure 3. Receiver operating characteristics curves for prediction of prolonged hospital admission (LOS > 14 days) for SEPSIS score and other risk prediction scores.**


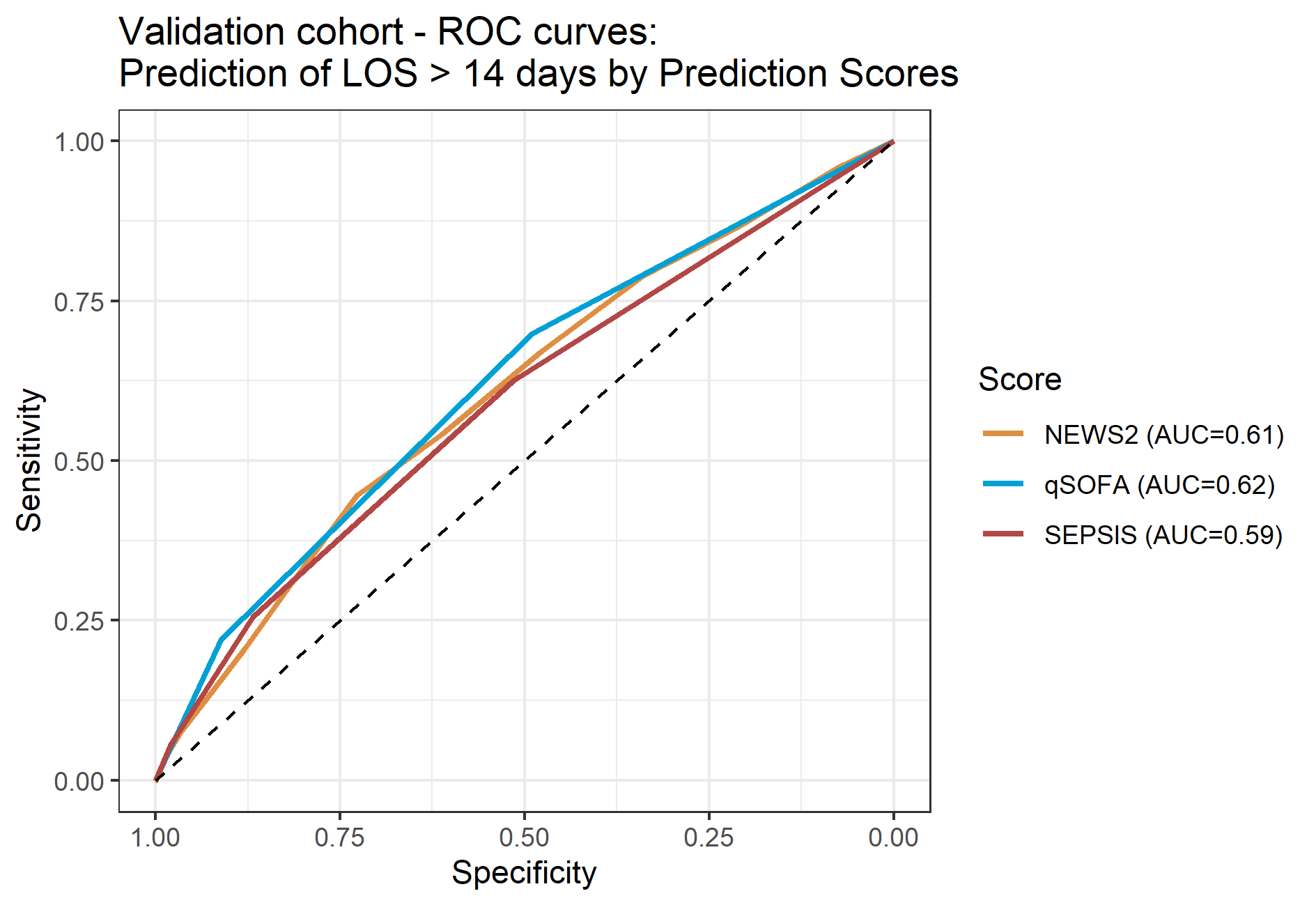


**Supplemental Figure 4. Bar graph of mortality risk by predictor score (low, medium, high) and score type.**


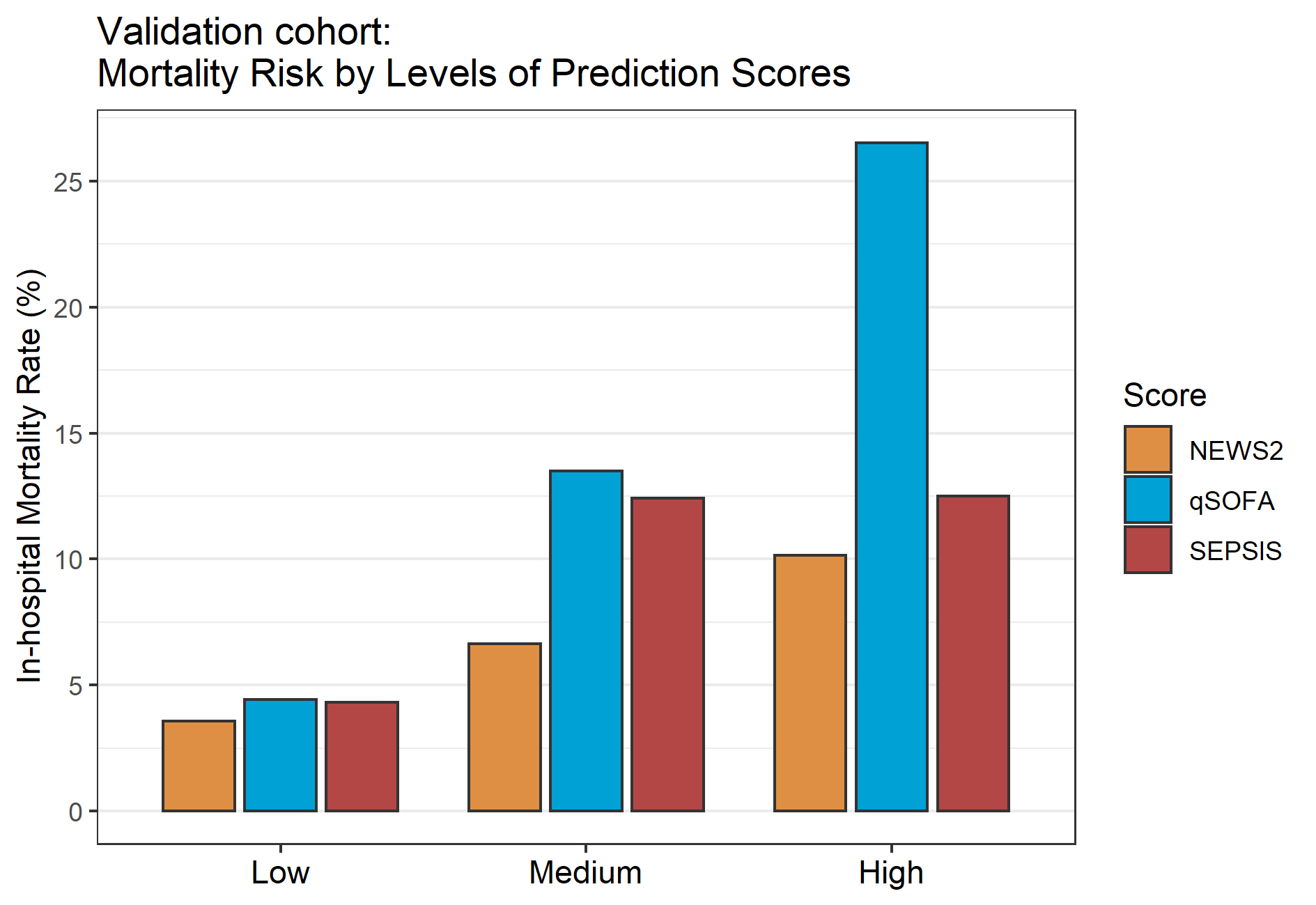

Supplement: S1 File — (DOCX) [file pone.0299473.s003.docx]
