# Supplementary material for: A simple electronic medical record-based predictors of illness severity in sepsis (sepsis) score
Source: PLoS One. 2024 Jun 26;19(6):e0299473. doi: 10.1371/journal.pone.0299473 (PMC11206954; doi:10.1371/journal.pone.0299473)
Supplement: S2 File — (DOCX) [file pone.0299473.s004.docx]

Supporting Information: Sepsis Score Summary

The SEPSIS Score is a new tool. This note summarizes the components of the score, which is openly available for the uses outlined in our manuscript and is not restricted or licensed to any institution or study personnel. It is designed for predicting in-hospital outcomes in patients hospitalized with sepsis, including mortality, ICU admission at 72 hours, and hospital length-of-stay.

In its development, we pre-selected predictors based on prior evidence, generalizability, simplicity, and adaptability. We did not use specific statistical approaches and elected to create the score without a need for change from baseline values.

The following variables and cut-offs were included in the score based on the standardized upper limits of normal at participating institutions.

For simplicity, we assigned each abnormal predictor (value above the cut-off) one point for the score, with a maximum total score of four, and a minimum score of zero. If a particular value was unavailable (e.g., there was no lactate ordered), then no point was given for that result type. We felt this was justifiable as the absence of a standard blood test is likely reflective of a patient with a lower severity of illness.

| **SEPSIS Score Component** | **Cutoff** |
| --- | --- |
| Creatinine | 125 µmol/L |
| Bilirubin | 35 µmol/L |
| Platelet Count | 100 x 10^9 |
| Lactate | 2 mmol/L |
